# Endothelial senescence in vascular diseases: current understanding and future opportunities in senotherapeutics

**DOI:** 10.1038/s12276-022-00906-w

**Published:** 2023-01-04

**Authors:** Yeaeun Han, Sung Young Kim

**Affiliations:** Department of Biochemistry, Konkuk University School of Medicine, Seoul, South Korea

**Keywords:** Senescence, Ageing, Drug discovery, Therapeutics, Cardiovascular diseases

## Abstract

Senescence compromises the essential role that the endothelium plays in maintaining vascular homeostasis, so promoting endothelial dysfunction and the development of age-related vascular diseases. Their biological and clinical significance calls for strategies for identifying and therapeutically targeting senescent endothelial cells. While senescence and endothelial dysfunction have been studied extensively, distinguishing what is distinctly endothelial senescence remains a barrier to overcome for an effective approach to addressing it. Here, we review the mechanisms underlying endothelial senescence and the evidence for its clinical importance. Furthermore, we discuss the current state and the limitations in the approaches for the detection and therapeutic intervention of target cells, suggesting potential directions for future research.

## Vascular disease: Improving vascular health by combatting ‘zombie’ cells

Cells that stop proliferating but remain metabolically active offer a promising therapeutic target for the treatment of age-related vascular diseases. Yeaeun Han and Sung Young Kim from Konkuk University School of Medicine in Seoul, South Korea, review the ways in which cellular senescence, the process by which damaged cells enter irreversible growth arrest, but stay alive and continue to secrete substances that damage surrounding tissues, contributes to blood vessel problems as people age. These so-called ‘zombie’ cells then fuel the development of atherosclerosis, hypertension, and other vascular disorders. Diagnostics that test for indicators of senescence affecting the lining of blood vessels could help reveal the severity of vascular damage. Drugs that remove or modify the function of senescent cells in blood vessels could help treat cardiovascular disease.

## Introduction

**Fig. 1:**
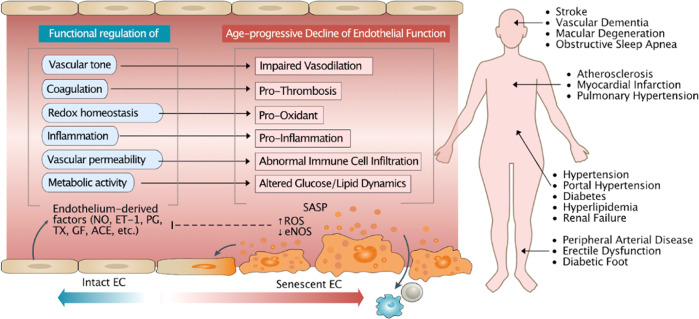
General overview of endothelial senescence and cardiovascular diseases (CVDs). Endothelial senescence is characterized by an age-associated decline in endothelial function, which includes the loss of control over vasodilation, blood coagulation, oxidative stress, inflammation, immune cell infiltration, and glucose and lipid dynamics mediated by endothelium-derived factors, such as nitric oxide (NO), endothelin-1 (ET-1), prostaglandin (PG), thromboxane (TX), endothelial growth factor (EGF) and angiotensin-converting enzyme (ACE). A reduction in NO, the master regulator of the endothelium, is one of the primary factors driving these changes. Other factors include increased reactive oxygen species (ROS) levels and senescence-associated secretory phenotype (SASP) acquisition. EC endothelial cell, eNOS endothelial nitric oxide synthase.

Age-related endothelial dysfunction, regarded as a prominent precursor to the development of cardiovascular diseases (CVDs)^1,2^, can be characterized by a shift toward a vasoconstrictive, proinflammatory and prothrombotic environment^3^, resulting in impaired regulation of vascular homeostasis. Endothelial dysfunction can be largely explained by endothelial senescence, which has been implicated in the development of various age-related CVDs, including stroke^4^, vascular dementia^5^, macular degeneration^6^, obstructive sleep apnea^7^, atherosclerosis^8,9^, myocardial infarction^10^, pulmonary hypertension^11–13^, hypertension^14^, diabetes^15,16^, renal failure^17^, peripheral arterial disease^18^, erectile dysfunction^19^ and diabetic foot^20^ (Fig. 1). The “geroscience hypothesis” aptly summarizes the idea that as aging is the main driver of multiple interrelated diseases, targeting key aging contributors may prevent the onset or mitigate the severity of multimorbidity^21^. Therefore, there are great clinical motivations and implications for research into endothelial senescence.

In this review, we discuss the key alterations found in endothelial senescence and its clinical potential in vascular diseases. The investigation into the clinical applications of endothelial senescence is characterized by two objectives: detecting and targeting senescence. By utilizing endothelial senescence as a molecular biomarker, it may be possible to describe the degree of endothelial dysfunction. Upon detection, senescent cells can be treated to reverse damage and recover endothelial function. Finally, we discuss the latest advances in detection approaches and in senotherapeutics, with a special focus on endothelial cells (ECs) and potential therapeutic applications of these advances to CVDs.

## Key pathways involved in endothelial senescence

Senescence serves as a blanket term that encompasses a wide range of states of stable cell cycle arrest states associated with various biological contexts and stimuli. Although such heterogeneity complicates the precise characterization or quantification of senescence, its implication in major age-related diseases makes it critical to investigate for a better understanding of their pathogenesis and the search for therapeutic venues. Rather than a simplistic approach in which various unique types of senescence are understood in the limited confines of generalized senescence, it may be useful to consider the individual features of a particular type of senescence in a defined physiological context.

While endothelial senescence has certain features that can be uniquely attributed to it, it shares several core mechanisms with other types of senescence. Endothelial senescence is triggered by a variety of senescence stressors that include replicative and oxidative stress, oncogenic activation^22^, telomere attrition^23^, DNA damage^24^, and mitochondrial dysfunction^25^. Mitochondrial dysfunction in senescent ECs can be characterized by decreased mitochondrial mass and alterations in the mitochondrial composition and electron transport chain (ETC)^26^. It has been demonstrated that the activity of complex IV and cytochrome c oxidase, components of the mitochondrial ETC, declines with age, and the resulting impairment increases mitochondrial oxidative stress^26^. In turn, oxidative stress can accelerate telomere shortening^27^ and induce DNA damage^24^ in ECs. Regardless of the type of stressor, senescent pathways converge on cell cycle arrest, which is primarily mediated by two main tumor suppressive pathways involving p53/p21^WAF1^ and/or p16^Ink4A^/RB^28^. Senescent cells acquire the senescence-associated secretory phenotype (SASP), which includes chemokines, cytokines, growth factors, and insoluble factors. While the SASP is heterogeneous and can vary by the type of cell and stimuli^29^, a unique feature of endothelial SASP is its characterization by regulators of arterial dysfunction, including increased levels of reactive oxygen species (ROS) and reduced nitric oxide (NO)^28^.

**Fig. 2:**
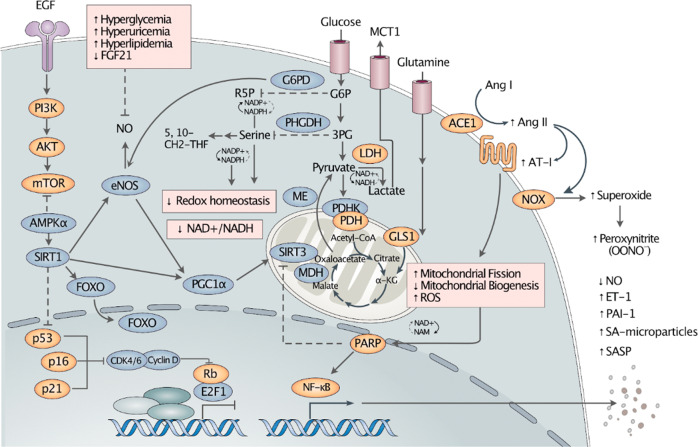
Altered pathways in endothelial senescence. We have marked proteins with activities that are believed to be reduced and increased in orange and blue, respectively. A prominent feature of endothelial senescence (ES) is impaired redox homeostasis with reduced antioxidant capacity. Senescent cells exhibit decreased NAD+/NADH levels, which may reduce ROS defense capacity. It has been suggested that potential alterations observed in ES can be summarized by the following: (1) active aerobic glycolysis driven by the activation of LDH and attenuated ME and MDH; (2) decreased PDHK activity leading to activated PDH, causing a shift to the TCA cycle; and 3) decreased PHGDH and G6PD activity leading to disruption of serine synthesis and of pentose phosphate pathway activity, respectively, and resulting in a decreased glutathione level and NADPH synthesis rate. Increased glutamine metabolism, along with an increased GLS1 level, may provide energy for senescent cells. Increased ACE, Ang II, and AT-1 activity can lead to reduced mitochondrial biogenesis and increased mitochondrial fission and ROS production. Impaired mitochondrial function can activate PARP to repair mitochondria. PARP regulates NF-κB, leading to SASP production. In ES, activated NOX may produce superoxide, which forms peroxynitrite with NO, resulting in a positive feedback loop that increases ROS and further decreases NO. EGF epidermal growth factor, PI3K phosphatidylinositol-3-kinase, mTOR mammalian target of rapamycin, AMPK adenosine monophosphate-activated protein kinase, SIRT sirtuin, FOXO forkhead box O, PGC1α peroxisome proliferator-activated receptor γ-coactivator 1α, CDK cyclin-dependent kinase, E2F1 E2F transcription factor 1, NF-κB nuclear factor-kappa B, PARP poly (ADP-ribose) polymerase, NAD nicotinamide adenine dinucleotide, NAM nicotinamide, NADH nicotinamide adenine dinucleotide hydrogen, NADP nicotinamide adenine dinucleotide phosphate, NADPH reduced nicotinamide adenine dinucleotide phosphate, NOX NADPH oxidase, AMP adenosine monophosphate, ADP adenosine diphosphate, ATP adenosine triphosphate, ROS reactive oxygen species, MDH malate dehydrogenase, α-KG α-ketoglutarate, GLS1 glutaminase 1, PDH pyruvate dehydrogenase, PDHK pyruvate dehydrogenase kinase, ME malic enzyme, LDH lactate dehydrogenase, PHGDH D-3-phosphoglycerate dehydrogenase, 5 10-CH2-THF 510-methenyltetrahydrofolate, 3PG 3-phosphoglycerate, G6P glucose 6-phosphate, G6PD glucose 6-phosphate dehydrogenase, R5P ribose 5-phosphate, MCT1 monocarboxylate transporter 1, ACE1 angiotensin-converting enzyme 1, ANG angiotensin, AT-1 angiotensin II type-1 receptor, NO nitric oxide, ET-1 endothelin-1, PAI-1 plasminogen activator inhibitor-1, SA senescence-associated, SASP senescence-associated secretory phenotype, eNOS endothelial nitric oxide synthase, FGF21 fibroblast growth factor 21.

Impaired redox homeostasis characterized by reduced NO production and increased oxidative stress is widely recognized as a key defining feature of endothelial senescence (Fig. 2). NO is a critical vasodilatory factor that has also been observed in reduction in endothelial dysfunction, owing to age-related impairment of endothelial nitric oxide synthase (eNOS) and decreased levels of the eNOS cofactors tetrahydrobiopterin (BH_4_) and L-arginine^30^. It has been reported that hyperglycemia^31^, hyperuricemia^32^, and hyperlipidemia^33^ inhibit eNOS production, while FGF21 has been shown to delay endothelial senescence by enhancing eNOS in mice^34^ and in a SIRT1-dependent manner in human umbilical vein endothelial cells (HUVECs)^34,35^. Diminished NO bioavailability may lead to impaired angiogenesis, reduced levels of peroxisome proliferator-activated receptor gamma coactivator 1 (PGC1α)^26^, and dysregulation in related metabolic processes such as the NAD+ biosynthetic pathway and NAD+-dependent protein deacetylase sirtuin-1 (SIRT1) in the sirtuin family^30^. Critical cellular sensors, adenosine monophosphate-activated protein kinase (AMPK), which senses the cell energy status, and mammalian target of rapamycin (mTOR), which senses the cell nutritional status, can modulate endothelial function and are altered during endothelial senescence^28^. In endothelial senescence, protein kinase B (Akt) has been reported to be constitutively activated and to promote cell arrest in a p53/p21-dependent manner through the inhibition of the transcription factor FOXO3a^36,37^. Inhibition of mTOR has been associated with enhanced endothelium-dependent dilation and higher levels of NO^38^. Another energy-sensitive metabolic sensor, sirtuin, plays an essential role in facilitating endothelial function through the regulation of the transcription factors FOXO, p53, and NFκB as well as in producing NO through the deacetylation of eNOS^28,39,40^. SIRT1 can further protect the endothelium by mediating the level of plasminogen activator inhibitor-1 (PAI-1), which can be increased via SIRT1 inhibition and is often increased in endothelial senescence and dysfunction^41^.

The renin/angiotensin system (RAS) is another key component of vascular tone regulation, which is altered in endothelial senescence. The altered RAS can be identified by increased angiotensin-converting enzyme 1 (ACE1), angiotensin II (Ang II), and angiotensin II type-1 receptor (AT-1), which have been shown to induce fibrosis, inflammation, and oxidative stress in aged mouse thoracic aorta^42^. In conjunction with increased endothelin-1^43^, a vasoconstrictor peptide produced by ECs^44,45^, the altered RAS exerts vasoconstrictive pressure on the vasculature^46^.

**Fig. 3:**
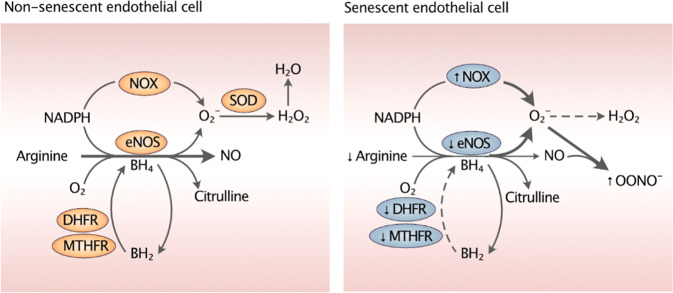
Overview of eNOS uncoupling in endothelial senescence. eNOS uncoupling occurs due to a reduction in the eNOS cofactors arginine and BH_4_ and is exacerbated by a decrease in the levels of DHFR and MTHFR, which are enzymes that regenerate BH_4_ by reducing BH_2_. eNOS and NOX uncoupling produces superoxide, which shows a higher affinity for NO than for its alternate scavenger, SOD, and ultimately leads to the production of OONO−, a potent oxidant. In addition to taking up NO for its own formation, OONO- oxidizes BH_4_ into BH_2_, reducing the eNOS cofactor. These mechanisms collaboratively decrease the NO availability during endothelial senescence. NADPH reduced nicotinamide adenine dinucleotide phosphate, NOX NADPH oxidase, eNOS endothelial nitric oxide synthase, *NO* nitric oxide, SOD superoxide dismutase, OONO- peroxynitrite, H_2_O_2_ hydrogen peroxide, H_2_O dihydrogen monoxide, BH_4_ tetrahydrobiopterin, BH_2_ 78-dihydrobiopterin, MTHFR methylenetetrahydrofolate reductase, DHFR dihydrofolate reductase.

Another cause of vasoconstrictive stress is derived from the uncoupling of eNOS (Fig. 3). The cause of eNOS uncoupling can be attributed to the limited availability of its cofactors such as L-arginine and BH_4_. Uncoupled eNOS and NADPH oxidase (NOX) can produce superoxide. While superoxide can be scavenged by superoxide dismutases, it has a higher affinity for NO and binds to it to form peroxynitrite (ONOO−), a toxic and reactive nitrogen species with strong oxidizing action^47^. ONOO−can oxidizes BH_4_ into 7,8-dihydrobiopterin (BH_2_) and damage zinc-thiolate clusters of eNOS, further driving eNOS uncoupling, ultimately increasing ROS and reducing NO^48^. It has been observed that dihydrofolate reductase (DHFR) and methylenetetrahydrofolate reductase (MTHFR), two enzymes responsible for the regeneration of BH_4_ from BH_2_, are impaired in senescence, further amplifying NO deficiency in a feed-forward loop. DHFR, a DNA-synthesizing enzyme, is attenuated in senescent cells^49^. Likewise, deficiency of MTHFR, an enzyme critical for the conversion of homocysteine to methionine, can lead to eNOS uncoupling in a BH_4_-dependent manner^50^. Interestingly, it has been reported that inhibition of s-adenosylhomocysteine hydrolase leads to senescence in HUVECs^51^. As evidenced by the role of the components of folate metabolism in endothelial senescence, it is possible that folate metabolism, which is altered in cancer and targeted for therapy, may also be critically involved in endothelial senescence. Further studies are needed to examine the key factors of folate metabolism, such as methylenetetrahydrofolate dehydrogenase, to elucidate the underlying mechanisms, which remain largely unknown.

Although much remains to be explored regarding the metabolic shifts in endothelial senescence, these shifts appear to play a crucial role in senescence. It is well-established that senescent cells are metabolically active^52,53^. However, the main source of energy in senescent ECs is still a matter of debate. Different studies of replicative senescence in HUVECs have reported conflicting results, showing that (1) glycolysis decreased with a decline in the expression of nuclear factor E2-related factor 2 (NRF2)^54^, a regulator of endothelial proliferation and glycolysis^55^; (2) the rate of glycolysis and the expression of glycolytic enzymes (hexokinase, LDH, aldolase, GAPDH, and pyruvate kinase) did not change significantly^56^; and (3) glycolysis was increased with increased expression of lactate dehydrogenase A and decreased expression of pyruvate dehydrogenase kinase 1-4^57^. While the latter two studies both noted an increase in glucose consumption and lactate production, the former study did not see a correlation between these two changes and suggested glutaminolysis is the alternative energy source for senescent ECs^56^. The same study showed that the inhibition of glutaminase (GLS) induced premature senescence in HUVECs. Supporting this finding, it was demonstrated that inhibition of GLS1 reduced the proliferative capacity and increased ROS in HUVECs^58^. Studies have shown that senescent cells are associated with lower levels of glucose 6-phosphate dehydrogenase (G6PD), which catalyzes the first step of the pentose phosphate pathway^59^, and of D-3-phosphoglycerate dehydrogenase (PHGDH), which catalyzes the committed step in de novo serine biosynthesis and regulates the critical steps of one-carbon metabolism^60^. G6PD and PHGDH play essential roles in redox homeostasis, mediating the production of NADPH, and the downregulation of these enzymes may significantly contribute to increased ROS in endothelial senescence^61,62^.

## Endothelial senescence in vascular diseases

Occupying the inner walls of blood vessels, ECs are subject to constant stresses from hemodynamic forces and various substances carried through the flowing blood^63^. Studies have shown that ECs positioned near arterial geometries such as bifurcations and curvatures in human tissue samples^23^ or otherwise exposed to disrupted blood flow^64,65^ undergo higher cell turnover and replicative senescence. Spikes in the partial oxygen pressure and the concentration of hormones and nutrients, which are notably increased during routine activities that cause abrupt metabolic changes in the body, such as exercising and eating, can lead to senescence. It has been demonstrated that high partial oxygen pressure can induce cellular senescence^66–68^, while culturing ECs in elevated levels of insulin^36^, glucose^69,70^ or uric acid^71^ showed reduced proliferative potential and accelerated onset of senescence. High levels of glucose or other reducing sugars can introduce advanced glycation end-products (AGEs) into the circulation^72^, and these AGEs can bind to and stimulate ECs, generating ROS^73^. Similarly, the levels of advanced oxidation protein products (AOPPs), which can be increased by AGEs, tend to be high in uremic patients and have been shown to induce senescence in HUVECs^74^. Other substances, such as circulating microparticles (MPs), which are shed from the membrane following a variety of stimuli and are circulated through the bloodstream^75^, can lead to endothelial dysfunction by mediating NO and ROS production^76,77^. It has been observed that exposure to MPs from acute coronary syndrome patients induces endothelial senescence through Ang 2, ACE1, and AT-1 of the altered RAS system^78^.

Endothelial senescence has been observed in multiple vascular organs, including the brain and the kidney, undermining critical functions of these organs and has thus been implicated in the development of CVDs. In the brain, cerebrovascular ECs comprise the blood‒brain barrier (BBB) and the neurovascular unit, and senescent phenotypes have been associated with increased BBB permeability and neurovascular uncoupling^79^. Proinflammatory SASP factors, such as interleukin 6 (IL-6) and interleukin 1β (IL-1β), are upregulated during aging and cerebrovascular diseases. Moreover, altered expression of proteases and protease regulators, such as matrix metalloproteinases (MMPs), can compromise tight junction integrity in the endothelium, all of which may contribute to a leaky BBB and ultimately to cerebrovascular diseases^79^. A comparison of kidneys in old and young mice showed that SASP factors contributing to glomerulosclerosis in old mice, such as PAI-1, IL-1β, IL-6, or MMPs^80^, were upregulated, and PAI-1 expressed by glomerular ECs may mediate crosstalk with podocytes to induce podocyte detachment and apoptosis^81^. The same study showed that endothelial senescence, as represented by high PAI-1 expression, was a negative prognostic marker for kidney transplantation, as recipients with PAI-1-positive kidneys were more likely to develop severe cases of glomerulosclerosis independent of other known clinical covariates^81^.

Various key features of endothelial senescence enhance susceptibility to the development of CVDs, where reduced capacity for replication and NO production and increased inflammation lead to pathophysiological consequences that can result in endothelial dysfunction^82^. Several studies have linked reduced replicative capacity and senescence in endothelial cells to reduced angiogenesis, such as impaired neovascularization, as observed in senescent HUVECs^83^, telomerase-deficient mice^84^ and SIRT1-silenced mice^85^. In turn, reduced angiogenic potential can lead to impaired wound healing and neovascularization, which are crucial to recovery from tissue damage, which may predispose patients to subsequent cardiovascular pathologies^86^. Proinflammatory cytokines contribute to vascular inflammation, which has been associated with atherogenesis and atherosclerosis^9^. Remodeling of the extracellular matrix (ECM) was shown to be disrupted in senescent ECs^82^. ECM degradation by MMPs, which are SASP factors, may result in arterial stiffening, exacerbated by reduced eNOS levels^87^, and an increase in the risk of hypertension^88^. Vasodilators and related factors, such as NO^89^, prostacyclin^90^, and eNOS^91^, have been observed to be decreased in senescent ECs. The ensuing endothelial dysfunction due to this reduction in NO bioavailability and loss of vascular tone has been implicated in cardiovascular events^92^.

Several genetic disorders further demonstrate the interconnectedness between senescence and CVD pathology. Hutchinson–Gilford progeria syndrome (HGPS) is a genetic disorder characterized by the accumulation of progerin, a mutant form of lamin A (LMNA)^93,94^, primarily in the vasculature, including the endothelium^95,96^. Similarly, Werner syndrome is caused by a mutation in the Werner syndrome helicase (WRN) gene, a DNA helicase gene that has been reported to play a critical role in endothelial homeostasis^97^. These diseases manifest as accelerated aging and premature death, often due to cardiovascular complications^98^, predominantly via atherosclerosis^99,100^. It has been observed that HGPS-derived ECs exhibited senescence traits^101,102^ and that the expression of progerin in ECs induced inflammation and senescence^103^, whereas deletion of LMNA in mice resulted in senescence-associated cardiomyopathy with increased SA-β-gal staining intensity and SASP protein levels^104^. Notably, deficient levels of G6PD, which has been shown to increase ROS and adhesion molecule levels in HUVECs^61^, has been identified as a risk factor for CVDs in elderly subjects^105,106^.

## Detection of endothelial senescence

Biomarkers frequently used to detect senescent ECs are features that have been broadly established across different types of cellular senescence. A common method to identify senescent cells is by examining their lysosomal content. SA-β-gal staining represents the activity of the lysosomal enzyme beta-galactosidase, which is detectable at the suboptimal cellular pH of 6.0^107^. Beta-galactosidase has been detected in CVD tissue samples from aged retinal blood vessels^108^, atherosclerotic plaques in aorta and coronary arteries^9,109^, and adipose tissue obtained from obese subjects^110^. Other biomarkers, such as cyclin-dependent kinase (CDK) inhibitors and telomere length^111^, have been implicated in the pathogenesis of various CVDs. Indeed, it has been reported that telomere length was negatively correlated with atherosclerotic grade in humans^112^, p16^Ink4A^ limited cell proliferation, and regeneration in response to pancreatic islet injury in mice^113^, and p53 was elevated in patients with congestive heart failure^114^ or hypertrophic cardiomyopathy^115^. Despite its broad use as a biomarker, increased SA-β-gal activity is not unique to senescent cells; it has been observed in cells induced to quiescence through serum starvation or confluence^116^. Similarly, while a high level of p16^Ink4A^ is a fairly well-established biomarker that is found consistently across aged human tissues^117^, it has also been observed in cells with inactivated RB, such as cancer cells^118^, but absent in cells undergoing certain forms of senescence in vitro^119,120^. To improve the detection of senescent cells, it is common to assess the levels of multiple markers, such as staining for SA-β-gal and γH2AX, which indicates DNA damage response activation^121^. Nevertheless, the lack of specificity of endothelial senescence biomarkers raises the question of whether the cells identified to date are truly senescent, and the search for a more precise characterization of senescence that caters to different cellular and physiological contexts remains an ongoing challenge.

Moreover, there are challenges to detecting senescent cells outside of a laboratory setting, which limit the clinical exploration of in vivo senescence in patients. The traditional methods of senescence detection rely on immunohistochemical staining of frozen or fixed tissues, which requires physical collection of tissues ex vivo. The need for in situ methods of measuring senescence has led to the development of liquid biopsy, a noninvasive approach that provides an alternative source of senescence identification. Components studied in liquid biopsy include circulating cells, extracellular vesicles, nucleosomes, and various glycoproteins and antigens^122^, which can serve as viable biomarkers. Circulating ECs, which include endothelial progenitor cells (EPCs) and blood outgrowth endothelial cells (BOECs), hold much promise as potential markers of endothelial senescence measured through liquid biopsy. It is well-established that EPCs actively contribute to cardiovascular homeostasis^123^ and are used as tools to study endothelial dysfunction^124^. There have been attempts to use EPCs for their vascular regenerative ability to treat related diseases, such as coronary artery disease^125^ and liver cirrhosis^126^. Accumulating evidence supports the idea that dysfunction and a reduction of EPCs are associated with cardiovascular risk factors such as aging, hypertension^127–129^, coronary artery disease^130^, and diabetes^128^. Senescent EPCs have been observed in preeclampsia^131^, and senescent circulating BOECs in smokers and chronic obstructive pulmonary disease patients^132^. Moreover, ECs secrete circulating microparticles, as observed in aging porcine coronary artery ECs^78^ and senescent mouse aortic ECs^133^. Known as endothelial microparticles (EMPs), these small extracellular vesicles (approximately 100–1000 nm) possess cellular reprogramming potential^134^ and procoagulant, proinflammatory tendencies^135^. Moreover, they play a crucial role in the paracrine induction of senescence and propagation of vascular aging^134,136^. An increase in EMP levels has been associated with various CVDs, such as stroke^137^, hypertension^138,139^, heart failure^140^, and acute coronary syndrome^78^, demonstrating their potential as a diagnostic biomarker. Other promising biomarkers to be utilized in liquid biopsy include AOPPs, which have been shown to induce senescence in HUVECs^74^, and AGEs, known to stimulate ECs and promote diabetes through oxidative stress^73^. While liquid biopsy can offer valuable information that spans various aspects of circulatory biology that can be observed and quantified across space and time, technical challenges remain in actual implementation of liquid biopsy. The low abundance of target components in the bloodstream, the lack of standardized protocols and platforms for analysis and interpretation, and high costs of setup^141,142^ present significant barriers to the research and technical development of liquid biopsy. Further research is needed to elucidate how potential markers for liquid biopsy can be used to detect senescence and to search for innovative ways to overcome the limitations.

## Therapeutic opportunities for endothelial senescence

Potential clinical implications of senescence have galvanized research into therapeutic approaches targeting senescent cells. Considerable progress has been made in two groups of senotherapeutics, senolytics, and senomorphics, in which a few of the compounds have entered clinical trials. Meanwhile, senescence immunotherapy has attracted interest for its potential in senotherapy and its efficacy in boosting the effectiveness of other treatments. We discuss the recent development and limitations of senotherapeutics and introduce several areas in which potential senotherapeutic targets have been identified.

Senolytics eliminate senescent cells by inducing apoptosis by targeting pathways such as BCL-2 family members, p53 and p38 MAPK^143^ (Table 1). Dasatinib is a tyrosine kinase inhibitor (TKI) that acts on a number of tyrosine kinases, including the Bcr-Abl and Src kinases. It is known for its initial efficacy in treating chronic myelogenous leukemia^144,145^ and has been repurposed as a senolytic^146^. It has been demonstrated that quercetin, a Bcl-xL-inhibiting flavonoid, and dasatinib, individually or in combination, are effective at reducing the number of senescent HUVECs^146,147^ and improving health and cardiac function in vivo^148–151^ and in idiopathic pulmonary fibrosis patients, where the treatment has led to enhanced physical functions and modest changes in SASP levels^152^. These promising results have led to a series of clinical trials focused on CVDs, including coronary artery diseases and idiopathic pulmonary fibrosis (Table 1). Fisetin has been reported to selectively induce apoptosis in HUVECs^153,154^, and three Bcl-xL inhibitors, A1331852, A1155463, and navitoclax, have been shown to induce apoptosis selectively in senescent HUVECs^153,154^ and in mice, with health improvements^155–158^. Recently, senolytics inhibiting glutaminolysis^159^, arginine metabolism^160^, and angiopoietin-like 2 (ANGPTL2)^161^, a SASP factor, have shown some potential. Nevertheless, much more evidence is needed to advocate for the clinical usage of senolytics. In 2018, the National Institute on Aging’s Interventions Testing Program^162^, considered the gold standard for testing longevity drugs, ran a series of studies on fisetin with genetically heterogeneous mice—as opposed to inbred mice commonly used in research—in three independent laboratories. The results showed that fisetin provided no benefits in extending lifespan or clearing senescent cells^163^. Similarly, despite numerous studies illustrating the in vitro and in vivo efficacy of quercetin, a study showed that quercetin induced cell death in both senescent and nonsenescent primary human coronary artery ECs, showing little specificity for senescence^164^.

**Table 1:** Summary of senotherapeutics and their in vitro and in vivo evidence in endothelial cells or cardiovascular diseases.

	Target mechanism	In vitro anti-senescence evidence	In vivo anti-senescence evidence	Clinical trial stage	Trial
Senolytics					
Quercetin	Inhibits PI3K/AKT, BCL-2 and Serpine activity	Induced apoptosis in IR-induced senescent HUVECs^146^	Reversed bleomycin-induced pulmonary fibrosis and reduced SASP marker levels in aged mice^149^ and Wistar rats^150^	Phase 2 in patients with Alzheimer’s disease	NCT04063124
					NCT04685590
				Phase 2 in patients with coronary artery disease	NCT04907253
Dasatinib	Inhibits tyrosine kinase activity	Induced apoptosis in IR-induced senescent human preadipocytes, and was much less effective on senescent HUVECs^146^	Exerted antidiabetic effects on older patients with type 2 diabetes mellitus in a retrospective cohort study^151^	Phase 2 with healthy participants	NCT04313634
Quercetin + Dasatinib		Induced apoptosis in IR-induced senescent HUVECs and preadipocytes^153^	1. Improved cardiac function and carotid vascular reactivity in old mice^146^ 2. Reduced hepatic steatosis in old mice^148^	Phase 2 with patients with chronic kidney disease	NCT02848131
				Phase 1 with patients with idiopathic pulmonary fibrosis	NCT02874989
Fisetin	Inhibits BCL-2 and PI3K/AKT	Selectively induced apoptosis in IR-induced senescent HUVEC^153^	Reduced senescence marker levels in multiple tissues in progeroid and old mice and restored tissue homeostasis, reduced age-related pathology and extended the lifespan of wild-type mice^155^	Phase 2 with patients with knee osteoarthritis	NCT04210986
					NCT04815902
				Phase 2 with patients with frailty	NCT03675724
					NCT03430037
					NCT04733534
A1331852, A1155463	Inhibits BCL-XL	A1331852 and AB1155463 selectively induced apoptosis in IR-induced senescent HUVECs and IMR90 cells^153^	Cleared senescent cholangiocytes and reduced SASP factor levels and attenuated liver fibrosis in mice^156^		
ABT263 (Navitoclax; UBX0101)	Inhibits BCL-2, BCL-XL and BCL-W	Induced apoptosis in IR-induced senescent HUVEC and IMR90c cells^154^	1.Cleared senescent cells in irradiated mice and normally aged mice^157^ 2. Cleared senescent cells and reduced atherogenesis in murine model of atherosclerosis^158^		
SSK1 (gemcitabine with an acetyl group and β-gal-responsive moiety)	Activates the p38 MAPK pathway	Selectively induced apoptosis in HUVECs^143^	Eliminated SA-β-gal-positive cells and senescence markers, attenuated lung fibrosis and reduced SASP factors in aged mice^143^		
BPTES	Inhibits glutaminase 1		Reduced the expression of p16^Ink4A^, KGA, and IL-6 mRNA and the KGA protein and ameliorated symptoms of diabetes, arteriosclerosis, and NASH in mice^159^		
ABH	Inhibits arginase 1	Reduced the number of senescent cells in bovine retinal endothelial cells^160^	Reduced senescence-associated diabetes-induced alterations (p16^Ink4A^, p21^WAF1^, p53, and IGFBP3) in diabetic mice^160^		
Senomorphics					
Rapamycin	Inhibits mTOR	Inhibited SASP acquisition in senescent pulmonary vascular endothelial cells from patients with chronic obstructive pulmonary disease^177^	1. Reduced cardiac inflammation and improved cardiovascular function in mice^178^ 2. Reversed age-related vascular dysfunction and improved arterial function in 30-month-old mice^179^		
Metformin	Inhibits IKK/NF-ƙB, activates AMPK	Reduced hyperglycemia-induced senescence and apoptosis in mouse microvascular endothelial cells^172^	Restored endothelial function and reduced inflammation in diabetic rats^176^	Phase 3 with people at high risk of developing diabetes	NCT00004992
					NCT00038727
Resveratrol	Inhibits NF-ƙB (an IĸB-kinase inhibitor), activates AMPK and SIRT1	1. Delayed the onset of senescence in human endothelial progenitor cells by increasing telomerase activity^170^ 2. Reduced oxidative reaction and inhibited senescence in endothelial progenitor cells^171^	1. Exerted cardioprotective effects on old senescence-accelerated mice^174^ 2. Reduced aorta media thickness inflammation, fibrosis and oxidative stress and inhibited arterial aging in mice^175^		
Ruxolitinib	Inhibits JAK1/2	Suppressed SASP acquisition in IR-induced senescent human preadipocytes and HUVECs and decreased inflammation in senescent cells^173^	Decreased SASP factor levels and enhanced physical activity in aged mice^173^		
FGF21	Activates SIRT1	Delayed replicative senescence and attenuated senescent phenotypes in HUVECs^35^	1. Protected against immunosenescence in mice^180^ 2. 2. Extended lifespan in transgenic mice with high levels of FGF21^181^		

IR irradiation, HUVEC human umbilical vein endothelial cell, SASP senescence-associated secretory phenotype, SIRT1 sirtuin-1, AMPK AMP-activated protein kinase, NASH nonalcoholic steatohepatitis.

Moreover, generations of cancer therapeutics have shown that even drugs once believed to be the most innovative, such as TKIs^165^, are inevitably associated with the risk of acquired resistance to therapy, potentially leading to a more intractable population of surviving tumor cells. Certainly, there is a need for consistent results from independent laboratories as well as longitudinal follow-up studies to observe the long-term effects of these drugs. The use of senolytics can result in far-reaching consequences on the body, as they may not be necessarily cell- or tissue-specific. Targets of senolytics include pathways that are involved in various biological processes in addition to senescence, and inhibition of one pathway may lead to the activation of another, as pathways are not entirely independent of one another and engage in crosstalk. Neglecting the interconnectedness of pathways may create the risk of unexpected adverse events.

Senomorphics are administered with the intention of attenuating the effects of detrimental senescent functions such as SASP, rather than removing senescent cells altogether. Senomorphics target certain components of the SASP factors or pathways that lead to the production of SASP factors, through NF-κB, JAK, SIRT1, and mTOR^166^. Ruxolitinib is a JAK1/2 inhibitor approved by the FDA to treat myelofibrosis^167^, and metformin and resveratrol have been shown to attenuate acquisition of the senescent phenotype via NF-ƙB inhibition^168,169^. The three aforementioned senomorphics showed in vitro and in vivo efficacy in ECs^170–172^ and aged mice^173–176^. Rapamycin, which inhibits mTOR, has demonstrated inhibitory effects on SASP acquisition and inflammation in senescent pulmonary vascular ECs^177^ as well as in mice, which showed cardiac improvement^178,179^. Furthermore, FGF21, a SIRT1 activator, reduced senescent phenotypes in HUVECs^35^, protected the immune system against senescence^180^, and extended the lifespan^181^ of mice. A caveat with senomorphics is the need for regular administration, as they do not eliminate the source of SASP, highlighting the need to investigate the long-term effects of senomorphics through longitudinal studies, as with senolytics. It is worth noting that while the overall benefits of senomorphics remain to be proven, studies have shown that regular consumption of certain senomorphics, such as metformin and D-glucosamine, has extended the lifespan of participants^166^.

Senescence immunotherapy mediates senescent clearance by taking advantage of the immune system. While this area of therapeutics needs further research and validation in endothelial senescence, it has been shown in liver ECs that SASP-induced endothelial phenotype plays a critical role in recruiting immunocytes in an endothelium-dependent manner^182^ and that the immune surveillance of senescent cells is mediated through two distinct EC pathways that depend on the NF-κB component RELA and inducible T-cell costimulator ligand (ICOSLG)^183^, suggesting possible targets for immunotherapy. Senescence immunotherapy may be an effective complement to existing drugs and treatments. For example, it can be utilized through antibodies targeting senescence-specific antigens to elicit immune responses against senescent cells and thus enhance the antitumor effects of cytotoxic drugs. A recent study reported on D9-HMSN@RSV, which consists of an antibody to CD9, a cell surface protein reported being upregulated in endothelial senescence and atherosclerosis^184^, conjugated to mesoporous silica nanoparticles (MSNs) to deliver rosuvastatin (RSV), an HMG-CoA reductase inhibitor^185^. CD9-HMSN@RSV produced positive results both in vitro and in vivo: it attenuated SASP acquisition, cleared senescent endothelial cells, and inhibited both the buildup of senescent plaque and the progression of atherosclerosis in mice. Another example of effective cotreatment involves the use of chimeric antigen receptor (CAR) T cells, where CAR T cells made to recognize urokinase-type plasminogen activator receptor (uPAR)—a cell surface protein widely upregulated in senescent cells—improved outcomes in lung adenocarcinoma and liver fibrosis in mice^186^. As certain SASP factors can facilitate immune responses, senescence-inducing therapy, followed by senescence immunotherapy, may be viable treatment for various refractory diseases by creating a more immunologically responsive environment. In an experiment with mouse models of pancreatic ductal adenocarcinoma, it was shown that vascular remodeling and endothelial activation mediated by therapy-induced senescence led to the accumulation of T cells in immunologically unresponsive tumors, potentiating PD-1 inhibitor immunotherapy^187^. Combined, these studies suggest that along with further research into endothelium-specific immunological features, the advent of senescence immunotherapy may be potentially applicable to endothelial senescence and CVDs.

## Future perspectives and conclusions

Certain compounds with a hormetic antiaging effect offer new possibilities for senotherapy. Gilbert syndrome (GS) is a genetic disorder characterized by mild hyperbilirubinemia caused by changes in *UGT1A1*, which encodes the enzyme required for the breakdown of bilirubin. Individuals with GS experience a lower incidence of CVDs, including coronary artery diseases and stroke^188,189^. While serum bilirubin has been reported to gradually and naturally increase with age^190^ and can be cytotoxic at high levels^191^, a moderately elevated level of bilirubin can be effectively antioxidant and anti-inflammatory, leading to improved endothelial function^192^. Interestingly, knockdown of biliverdin reductase A (BLVRA), an enzyme that converts biliverdin to bilirubin, induced senescence, whereas its overexpression restored certain nonsenescent features in senescent cells^193^. Although the narrow therapeutic window necessitates further research into its clinical viability, bilirubin is a promising candidate for endothelial senescence therapeutics.

Another promising venue worthy of further research lies in targeting nucleocytoplasmic trafficking (NCT). A study of postmitotic cells in rats revealed the presence of extremely long-lived proteins associated with nuclear pore complex (NPC) over the observational period^194^, a striking contrast to the majority of proteins, which are usually turned over in a matter of days^195^. Along with nuclear transport receptors (NTRs) and Ran, NPC comprises the NCT, a specialized machinery involved in the transport of macromolecules, such as signaling proteins and messenger ribonucleoproteins (mRNPs)^196^. It has been reported that a set of genes associated with NCT—as well as genes associated with the machinery responsible for transport of mRNA, the transcription-export (TREX) complex—is downregulated in various types of senescence, including endothelial senescence^196,197^. A recent study showed that senescent lens epithelial cells showed concomitant downregulation of a group of NCT genes and failure of the nuclear translocation of BLVRA and NRF2^198^, suggesting a possible role for NCT in the impaired delivery of these two proteins. A recent study into the disruption of the NCT machinery in fibroblasts revealed a phenotype resembling replicative senescence, indicating that several potential senescence drivers in the NCT may be leveraged for senescence therapy^199^. Further studies are needed to understand the mechanisms and validate the utility of NCT and TREX factors.

Still, whether or not the elimination of senescent cells is indeed beneficial is yet to be determined. In a study of stress-induced senescence in pulmonary microvascular ECs in mice, senescent cells were removed via the senolytic agent ABT263, leading to a reversal in blood vessel remodeling and pulmonary arterial hypertension^12^. However, a study of p16^Ink4A^–Cre knockin mice presented contrasting results. The genetic elimination of cells, mainly liver vascular ECs, with high levels of p16^Ink4A^ with an ablator line (Rosa26-DTA) caused blood tissue barrier disruption, liver fibrosis, and health deterioration in some mice, as the removed senescent cells were not replaced^200^. Additionally, a study on the effect of navitoclax in atherosclerosis presented contradictory results in which the number of senescent cells and the degree of atherogenesis were reduced in culture but the senescence marker levels were not reduced in vivo^201^. Moreover, many of the studies of EC senescence tend to be based on HUVECs, at least utilized in the preliminary study. Although a cost-effective and accessible option, HUVECs are collected from the immune-privileged environment of fetal tissues^202^ and therefore harbor several fundamental differences compared to adult endothelial cells^203^. These differences present the possibility that results from HUVECs may not necessarily be accurate representations of adult vascular pathophysiology^203^ and suggest that we may need to find a cell line that better represents in vivo conditions.

Much remains to be explored and further validated in endothelial senescence research. The conflicting results from senescence studies highlight the need to refine the biomarkers to each pathophysiological context, as no single marker can be used to identify senescent cells conclusively. Most likely, collecting a set of markers—those that are universal and those that are more specific—and adjusting them to fulfill different purposes will be necessary. For example, encapsulation of navitoclax, a senolytic associated with thrombocytopenia^204^, with beads coated with galactooligosaccharides, which respond to β-galactosidase activity, has led to the clearance of senescent cells and showed low toxicity^205^. This emphasizes the importance of the detection of senescent cells and the delivery of senotherapeutics. Senotherapeutics targeting senescent ECs not only target age-associated deterioration but also lead to improvements in cardiovascular function, which are areas of great interest in view of the burgeoning aging population worldwide. In summary, further exploration into the molecular mechanisms and precise characterization of endothelial senescence and how these mechanisms can be manipulated for therapeutics could have a meaningful impact on developing treatments for vascular diseases.
